# Comprehensive Analysis to Improve the Validation Rate for Single Nucleotide Variants Detected by Next-Generation Sequencing

**DOI:** 10.1371/journal.pone.0086664

**Published:** 2014-01-29

**Authors:** Mi-Hyun Park, Hwanseok Rhee, Jung Hoon Park, Hae-Mi Woo, Byung-Ok Choi, Bo-Young Kim, Ki Wha Chung, Yoo-Bok Cho, Hyung Jin Kim, Ji-Won Jung, Soo Kyung Koo

**Affiliations:** 1 Division of Intractable Diseases, Center for Biomedical Sciences, National Institute of Health, Chungcheongbuk-do, South Korea; 2 Macrogen Inc., Gasan-dong, Seoul, South Korea; 3 Department of Neurology, Samsung Medical Center, Sungkyunkwan University, School of Medicine, Seoul, South Korea; 4 Department of Biological Science, Kongju National University, Gongju, South Korea; 5 Department of Biological Sciences, KAIST, Yuseong-gu, Daejeon, South Korea; University of Torino, Italy

## Abstract

Next-generation sequencing (NGS) has enabled the high-throughput discovery of germline and somatic mutations. However, NGS-based variant detection is still prone to errors, resulting in inaccurate variant calls. Here, we categorized the variants detected by NGS according to total read depth (TD) and SNP quality (SNPQ), and performed Sanger sequencing with 348 selected non-synonymous single nucleotide variants (SNVs) for validation. Using the SAMtools and GATK algorithms, the validation rate was positively correlated with SNPQ but showed no correlation with TD. In addition, common variants called by both programs had a higher validation rate than caller-specific variants. We further examined several parameters to improve the validation rate, and found that strand bias (SB) was a key parameter. SB in NGS data showed a strong difference between the variants passing validation and those that failed validation, showing a validation rate of more than 92% (filtering cutoff value: alternate allele forward [AF]≥20 and AF<80 in SAMtools, SB<–10 in GATK). Moreover, the validation rate increased significantly (up to 97–99%) when the variant was filtered together with the suggested values of mapping quality (MQ), SNPQ and SB. This detailed and systematic study provides comprehensive recommendations for improving validation rates, saving time and lowering cost in NGS analyses.

## Introduction

Next-generation sequencing (NGS) provides cheap and reliable large-scale DNA sequencing [1]. NGS has recently been introduced as an effective tool for genetic screening and many recent publications have described new disease-causing variants discovered by whole exome sequencing [2]. NGS techniques generate large numbers of DNA sequence variants, which must be analyzed and filtered to find candidates for disease causation. However, NGS-based variant detection is prone to erroneous calls and generates low-interest variants in the form of genotype false-positives.

NGS data can contain errors due to technological and biological biases, as well as systematic problems [3]–[9]. Errors can arise from biases in target enrichment [3], sequence effects or base calling sequence errors [4]–[7], uncertainties in read alignments [6], [7], batch effects [7] or platform-specific mechanistic problems [8], [9]. The choice of software tool has a clear impact on the identified variants [10]. Variant-detection algorithms of software tools are other important sources of false-positive calls in NGS data [11]. A critical step in detecting variants from NGS is filtering the putative variants called using analysis algorithms and parameters.

NGS variant-detection algorithms such as SAMtools [12] and GATK [13] produce multiple parameters for each variant call, including coverage, SNP quality (SNPQ), mapping quality (MQ), and strand bias (SB). Variant-detection algorithms infer the actual nucleotide information from obtained florescence-intensity data for each aligned read. This information is then assigned as SNPQ, which is a measure of Phred-scale quality scores to each base call. MQ is a measure of the uncertainty that a read is mapped to the proper genomic position. SB can occur where there is a highly unequal distribution of forward vs. reverse directions in aligned reads. However, it is unclear how these parameters should be interpreted with regard to whether a variant call is correct. Furthermore, multiple parameters are generated for each variant call, and thus one cannot simply rank or prioritize variants using the values. For this reason, researchers often rely on personal experience and arbitrary filtering thresholds to select variants. Also, researchers prefer variants identified using all algorithms (common variants) than variants identified by individual programs (caller-specific variants) to reduce the false discovery rate (FDR) [10]. However, there is a lack of methods for assigning a single accuracy estimate to individual variants. A consensus approach for confident putative variant analyses would enable prioritizing and selecting variants in a robust manner. A high validation rate would not only reduce the cost and labor in experimental validation of NGS data, but also avoid reporting false discoveries in the literature or public databases.

In this study, we used Sanger sequencing with single nucleotide variants (SNVs) detected by NGS using our own experimental data and systematically examined the validation rate according to total read depth (TD) and SNPQ. We evaluated several major parameters that affect variant calling, and provide some guidelines for choosing appropriate parameters for variant calls from NGS data.

## Materials and Methods

### Generation of Sequence Data

The study included 30 Korean Charcot-Marie-Tooth disease patients with whole exome data available from previous studies [14], [15]. Written informed consent was obtained from all Korean participants according to the protocol approved by the Institutional Review Board of Ewha Woman’s University (Mokdong Hospital) and Korea National Institute of Health (KNIH).

The whole exome was captured from genomic DNA using the Human SeqCap EZ Human exome library v2.0 (Roche-NimbleGen, Madison, WI), and NGS was performed using the Solexa GAIIx Genome Analyzer (Illumina, San Diego, CA). Sequencing libraries were prepared following the standard Illumina library-preparation protocol for paired-end 76-bp reads. Raw ‘fastq’ files for both reads were generated and used for the alignment processes.

### Exome Data Analysis

Raw sequence reads were first mapped to the human genome with the reference sequence of UCSC assembly hg19 (NCBI build37.1) using the BWA program (http://bio-bwa.sourceforge.net/). Any reads not across the targeted exonic regions were filtered out. After creating a consensus sequence from the BWA mapping (a BAM file), the variant calling process was performed using the two most popular calling algorithms, SAMtools (http://samtools.sourceforge.net/) and GATK (http://www.broadinstitute.org/gatk/), following the guidelines presented in the user manual. SNP data were generated using the mpileup utility of SAMtools. SNV data were also generated using the Unified Genotyper of GATK as the tool to call variation. Targeted realignment and base recalibration were performed using GATK. Then, the variants were annotated with the ANNOVAR program (http://www.openbioinformatics.org/annovar/).

### Analysis Pipeline for SNV Detection

**Figure 1:**
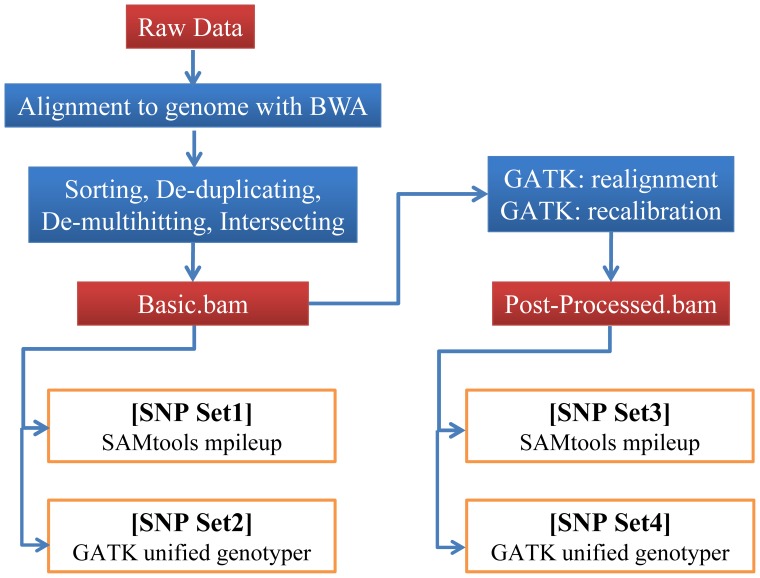
Pipelines for calling single nucleotide variants (SNVs). SNVs were called in four sets, based on SAMtools: mpileup (SNP set1 and SNP set3) and GATK: unified genotyper (SNP set2 and SNP set4). The numbers of reads and SNPs for individual steps are given for one exome-seq data set, generated using a Solexa GAIIx Genome Analyzer.

The analysis pipeline for detecting SNVs generally consisted of the following steps: (1) pre-processing, (2) mapping the reads to the reference genome, (3) post-processing, (4) calling SNVs and (5) filtering. We first trimmed the adapter sequences with an in-house script, applied BWA (ver. 0.5.9) to map reads using default parameters (excluding the −q 20 option), used Picard tools (ver. 1.8.5) to sort and mark duplicates and intersected the reads within target regions using BEDtools (ver. 2.17.0). We denoted the BAM file prepared using this pipeline as the basic BAM file. In the filtering step for SAMtools, we used “perl vcfutils.pl varFilter” with a “−D 1000” option and other parameters by default and named the variant results as SNP set1. In the filtering step for GATK, we used the GATK-implemented variant recalibrator with known variant data (e.g., hapmap 3.3, 1000G omni 2.5 and dbSNP 135) and named the variant results as SNP set2. In addition, we realigned and recalibrated the basic BAM file with GATK (ver. 1.6) before the variant-calling step, to improve the initial mapping results before variant calling. After the integration of these two steps, post-processed bam files were generated for SAMtools and GATK, and we generated two types of variant sets (SNP set3 and SNP set4, respectively) in the same manner as the analysis of the basic BAM file (Figure 1).

### Distribution of Variants According to TD and SNPQ Values

We categorized the SNVs called by SAMtools and/or GATK according to TD and SNPQ values. The TD values were divided into five categories: TD<5, 5≤TD<10, 10≤TD<20, 20≤TD<40 and 40≤TD. The SNPQ values were divided into nine categories for each tool. For the SAMtools analysis, they were divided as follows: SNPQ<10, 10≤SNPQ<20, 20≤SNPQ<30, 30≤SNPQ<40, 40≤SNPQ<60, 60≤SNPQ<80, 80≤SNPQ<100, 100≤SNPQ<200 and 200≤SNPQ. For the GATK analysis, they were divided as follows: SNPQ<20, 20≤SNPQ<50, 50≤SNPQ<100, 100≤SNPQ<150, 150≤SNPQ<200, 200≤SNPQ<3000, 300≤SNPQ<500, 500≤SNPQ<1000 and 1000≤SNPQ. We matched the categories of SNPQ as consensus values between SAMtools and GATK.

### Sanger Sequencing Analysis for Selected SNVs

In total, 348 non-synonymous variants were selected randomly for validation by Sanger sequencing. The selected SNVs were generally well-distributed in 45 TD and SNPQ categories (Table 1). The variants were genotyped by PCR amplification using 50 ng of DNA, followed by Sanger sequencing using an ABI 3730 automatic genetic analyzer (Applied Biosystems, Foster City, CA). The sequence reads were analyzed using the Sequencer software package (Gene Codes Corp., Ann Arbor, MI). Reactions were successful for all loci, after which these loci were compared to the results generated using NGS data.

**Table 1:** Validation rates according to TD and SNPQ categories in 4 analysis pipelines.

SNP Set1						
Category	TD<5	5≤TD<10	10≤TD<20	20≤TD<40	40≤TD	Total
**SNPQ<10**	5/9 (55.6%)	5/13 (38.5%)	5/10 (50.0%)	4/7 (57.1%)	0/4 (00.0%)	19/43 (44.2%)
**10≤SNPQ<20**	**7/8 (87.5%)**	5/8 (62.5%)	4/12 (33.3%)	4/12 (33.3%)	1/5 (20.0%)	21/45 (46.7%)
**20≤SNPQ<30**	4/8 (50.0%)	**10/12 (83.3%)**	3/12 (25.0%)	0/9 (00.0%)	2/5 (40.0%)	19/46 (41.3%)
**30≤SNPQ<40**	7/9 (77.8%)	5/7 (71.4%)	3/9 (33.3%)	1/7 (14.3%)	0/3 (00.0%)	16/35 (45.7%)
**40≤SNPQ<60**	**6/6 (100.0%)**	6/9 (66.7%)	5/10 (50.0%)	4/12 (33.3%)	2/5 (40.0%)	23/42 (54.8%)
**60≤SNPQ<80**	**1/1 (100.0%)**	6/9 (66.7%)	**6/7 (85.7%)**	6/8 (75.0%)	**5/6 (83.3%)**	24/31 (77.4%)
**80≤SNPQ<100**	**2/2 (100.0%)**	**7/8 (87.5%)**	**10/10 (100.0%)**	**8/10 (80.0%)**	6/8 (75.0%)	33/38 (86.8%)
**100≤SNPQ<200**	**2/2 (100.0%)**	**6/6 (100.0%)**	**13/13 (100.0%)**	**10/10 (100.0%)**	**6/7 (85.7%)**	37/38 (97.4%)
**200≤SNPQ**	0/0 (–)	0/0 (–)	**10/10 (100.0%)**	**9/10 (90.0%)**	**10/10 (100.0%)**	29/30 (96.7%)
**Total**	34/45 (75.6%)	50/72 (69.4%)	59/93 (63.4%)	46/85 (54.1%)	32/53 (60.4%)	221/348 (63.5%)
**SNP Set2**						
**Category**	**TD<5**	**5≤TD<10**	**10≤TD<20**	**20≤TD<40**	**40≤TD**	**Total**
SNPQ<20	1/2 (50.0%)	1/2 (50.0%)	0/1 (00.0%)	0/0 (–)	0/0 (–)	2/5 (40.0%)
20≤SNPQ<50	11/15 (73.3%)	7/10 (70.0%)	2/9 (22.2%)	1/6 (16.7%)	0/0 (–)	21/40 (52.5%)
50≤SNPQ<100	7/9 (77.8%)	9/13 (69.2%)	1/6 (16.7%)	0/7 (00.0%)	0/4 (00.0%)	17/39 (43.6%)
100≤SNPQ<150	**7/8 (87.5%)**	**16/17 (94.1%)**	8/14 (57.1%)	3/10 (30.0%)	0/4 (00.0%)	34/53 (64.2%)
150≤SNPQ<200	0/0 (–)	**5/6 (83.3%)**	6/8 (75.0%)	4/8 (50.0%)	1/10 (10.0%)	16/32 (50.0%)
200≤SNPQ<300	0/0 (–)	8/11 (72.7%)	**15/16 (93.8%)**	8/13 (61.5%)	1/7 (14.3%)	32/47 (68.1%)
300≤SNPQ<500	0/0 (–)	**3/3 (100.0%)**	**11/11 (100.0%)**	**17/21 (81.0%)**	2/11 (18.2%)	33/46 (71.7%)
500≤SNPQ<1000	0/0 (–)	0/0 (–)	**7/7 (100.0%)**	**14/16 (87.5%)**	15/24 (62.5%)	36/47 (76.6%)
1000≤SNPQ	0/0 (–)	0/0 (–)	0/0 (–)	**5/5 (100.0%)**	**19/22 (86.4%)**	24/27 (88.9%)
Total	26/34 (76.5%)	49/62 (79.0%)	50/72 (69.4%)	52/86 (60.5%)	38/82 (46.3%)	215/336 (64.0%)
**SNP Set3**						
**Category**	**TD<5**	**5≤TD<10**	**10≤TD<20**	**20≤TD<40**	**40≤TD**	**Total**
**SNPQ<10**	**6/7 (85.7%)**	7/10 (70.0%)	1/6 (16.7%)	3/8 (37.5%)	0/4 (00.0%)	17/35 (48.6%)
**10≤SNPQ<20**	5/7 (71.4%)	6/11 (54.5%)	5/13 (38.5%)	2/6 (33.3%)	0/3 (00.0%)	18/40 (45.0%)
**20≤SNPQ<30**	6/8 (75.0%)	2/4 (50.0%)	3/8 (37.5%)	3/5 (60.0%)	0/3 (00.0%)	14/28 (50.0%)
**30≤SNPQ<40**	3/6 (50.0%)	**8/10 (80.0%)**	3/10 (30.0%)	0/2 (00.0%)	0/2 (00.0%)	14/30 (46.7%)
**40≤SNPQ<60**	**7/7 (100.0%)**	**8/8 (100.0%)**	4/7 (57.1%)	3/8 (37.5%)	**4/5 (80.0%)**	26/35 (74.3%)
**60≤SNPQ<80**	**2/2 (100.0%)**	9/13 (69.2%)	**8/9 (88.9%)**	**4/4 (100.0%)**	1/3 (33.3%)	24/31 (77.4%)
**80≤SNPQ<100**	**4/4 (100.0%)**	2/3 (66.7%)	**12/12 (100.0%)**	**9/10 (90.0%)**	**6/6 (100.0%)**	33/35 (94.3%)
**100≤SNPQ<200**	**1/1 (100.0%)**	**6/6 (100.0%)**	**12/12 (100.0%)**	**14/15 (93.3%)**	**9/10 (90.0%)**	42/44 (95.5%)
**200≤SNPQ**	0/0 (–)	0/0 (–)	**7/7 (100.0%)**	**8/9 (88.9%)**	**10/10 (100.0%)**	25/26 (96.2%)
**Total**	34/42 (81.0%)	48/65 (73.8%)	55/84 (65.5%)	46/67 (68.7%)	30/46 (65.2%)	213/304 (70.1%)
**SNP Set4**						
**Category**	**TD<5**	**5≤TD<10**	**10≤TD<20**	**20≤TD<40**	**40≤TD**	**Total**
**SNPQ<20**	1/2 (50.0%)	**1/1 (100.0%)**	0/2 (00.0%)	0/1 (00.0%)	0/1 (00.0%)	2/7 (28.6%)
**20≤SNPQ<50**	10/13 (76.9%)	7/10 (70.0%)	2/6 (33.3%)	1/6 (16.7%)	0/3 (00.0%)	20/38 (52.6%)
**50≤SNPQ<100**	**8/10 (80.0%)**	11/14 (78.6%)	2/7 (28.6%)	1/8 (12.5%)	0/6 (00.0%)	22/45 (48.9%)
**100≤SNPQ<150**	**7/8 (87.5%)**	**14/15 (93.3%)**	8/14 (57.1%)	2/11 (18.2%)	0/5 (00.0%)	31/53 (58.5%)
**150≤SNPQ<200**	0/0 (–)	**5/6 (83.3%)**	**5/6 (83.3%)**	2/4 (50.0%)	1/5 (20.0%)	13/21 (61.9%)
**200≤SNPQ<300**	0/0 (–)	10/13 (76.9%)	**14/15 (93.3%)**	**10/12 (83.3%)**	1/10 (10.0%)	35/50 (70.0%)
**300≤SNPQ<500**	0/0 (–)	**1/1 (100.0%)**	**12/12 (100.0%)**	**17/18 (94.4%)**	2/8 (25.0%)	32/39 (82.1%)
**500≤SNPQ<1000**	0/0 (–)	0/0 (–)	**7/7 (100.0%)**	**14/16 (87.5%)**	15/21 (71.4%)	36/44 (81.8%)
**1000≤SNPQ**	0/0 (–)	0/0 (–)	0/0 (–)	**5/5 (100.0%)**	**19/22 (86.4%)**	24/27 (88.9%)
**Total**	26/33 (78.8%)	49/60 (81.7%)	50/69 (72.5%)	52/81 (64.2%)	38/81 (46.9%)	215/324 (66.4%)

### Adjustment of Analysis Parameters to Improve the Validation Rate

Additional analysis parameters for filtering variants, including MQ score, genotype quality (GQ) score, alteration read percentage and SB, were assessed and compared to those established during validation. SB is observed either when the majority of sequence reads originate from only one DNA strand or when variant bases occur preferentially on one strand compared to the other. In the SAMtools program, the percent of alternate allele forward (AF) was taken to be the SB value.

## Results

### Validation of SNVs by Sanger Sequencing

To check the validation rate of variants by Sanger sequencing, we used one exome-seq data generated from an Illumina GA II and focused on non-synonymous variation based on potential functional importance. To avoid sample-specific bias, we previously examined differences in the number or SNPQ value patterns of non-synonymous SNVs among 30 exome-seq data sets [14], [15], and observed no significant differences in distribution patterns of SNPQ value (Figure S1).

Starting with SNP set1 (SAMtools, Basic), we selected and sequenced 348 SNPs, which were categorized according to TD and SNPQ. Primers were designed for each variant site, and traditional Sanger sequencing was used to sequence the corresponding PCR products. The overall validation rate was very low (63.5%, 221/348; Table 1). When we analyzed SNP set2 (GATK, Basic), the validation rate was similar to that of SNP1 (64%, 215/336). After the integration of realignment and recalibration, the validation rates of SNP set3 (SAMtools, recalibration) and SNP set4 (GATK, recalibration) increased marginally, to 70.1% (213/304) and 66.4% (215/324), respectively (Table 1). Several variants, as detected by NGS and confirmed (or not) by conventional Sanger sequencing, are shown in Figure S2. The data sets of variants generated from four pipelines were shown in the Supporting Information (Dataset S1, S2, S3 and S4).

### Distribution of Validation Ratio According to SNPQ and TD

**Figure 2:**
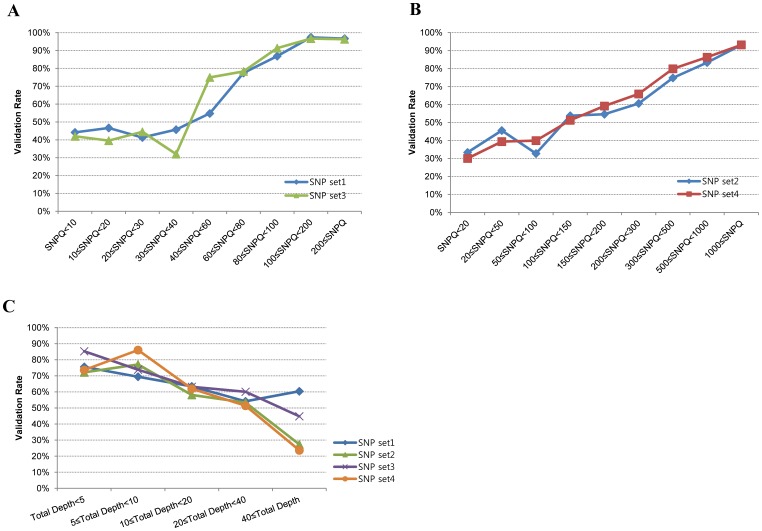
Distribution of validation rate according to SNP quality (SNPQ) and total read depth (TD). (A) Validation rate of SNPQ for SAMtools (SNP set1 and SNP set3). (B) Validation rate of SNPQ for GATK (SNP set2 and SNP set4). (C) Validation rate of TD for SNP set1–4.

In early applications using SAMtools:pileup to call variants, a cutoff value of 20 for SNPQ was suggested. Because SNPQ scores of variants called by mpileup and pileup are quite similar, it has been thought that 20 can also generally be used for variant-calling by SAMtools:mpileup. However, appropriate cutoff values for SNPQ to filter putative SNVs have not been established. To determine appropriate cutoffs for SAMtools:mpileup (SNP set1 and SNP set3) and GATK:unifiedgenotyper (SNP set2 and SNP set4), we first checked the correlation between validation rate and SNPQ value. Coincident with the definition of Phred-scale value, the validation rate of SNPs with high SNPQ values were positively correlated with SNPQ value (Figure 2A and 2B). We suggest that an SNPQ cutoff of 80 is more appropriate than 20 because when this value was applied to call variants using SAMtools:mpileup the cutoff showed a validation rate of more than 80% by Sanger sequencing. In the case of GATK, SNPQ values greater than 300 had a validation rate of more than 80% by Sanger sequencing. As averages for entire categories, the validation rates increased to 87.7% and 83.6% when SNVs were filtered with 40 in SAMtools and 300 in GATK (Table 1).

SNPs with high TD are generally considered more reliable than those with low TD. Thus, we checked the correlation between TD and the validation rate of SNPs. Contrary to our expectations, TD was negatively correlated with validation rate (Figure 2C). This result was caused by the discrepancy between TD and SNPQ values. For example, only high TD variants with high SNPQ were validated. Accordingly, the SNPQ value is more important than the TD of SNVs.

### Common Variant Set with an Improved Validation Rate

**Figure 3:**
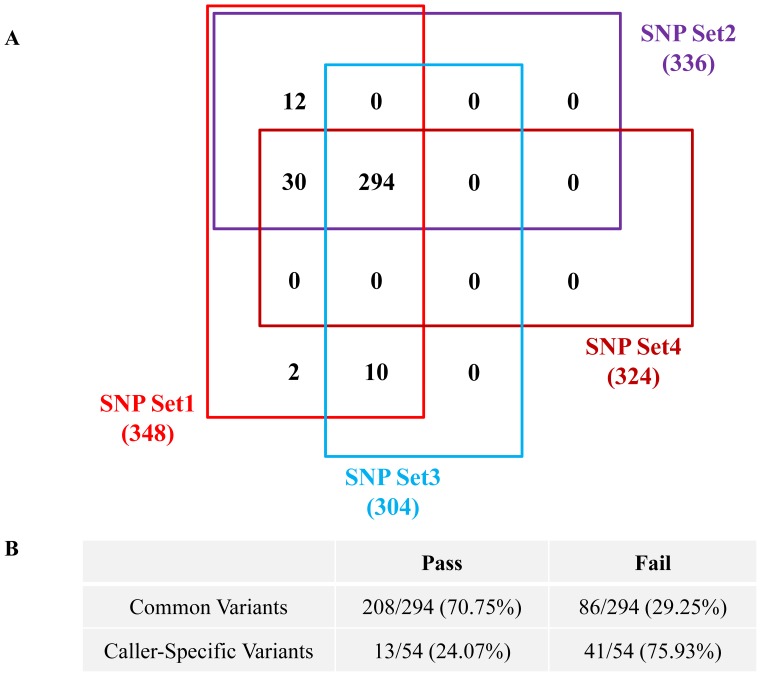
Diagram and validation rate of common variants. (A) The diagram of common variants among the four types of SNP sets. (B) The validation rates of common variants and caller-specific variants.

We further checked whether the common variants called by both SAMtools and GATK were more accurate than caller-specific variants. Of 348 variants, 294 were common and 54 were caller-specific, and their validation rates were 70.75% (208/294) and 24.07% (13/54), respectively (Figure 3). Thus, the selection of common variants called by both algorithms helps to improve validation rates.

### Evaluation of SB as a Key Parameter for Improving Validation Rate

**Figure 4:**
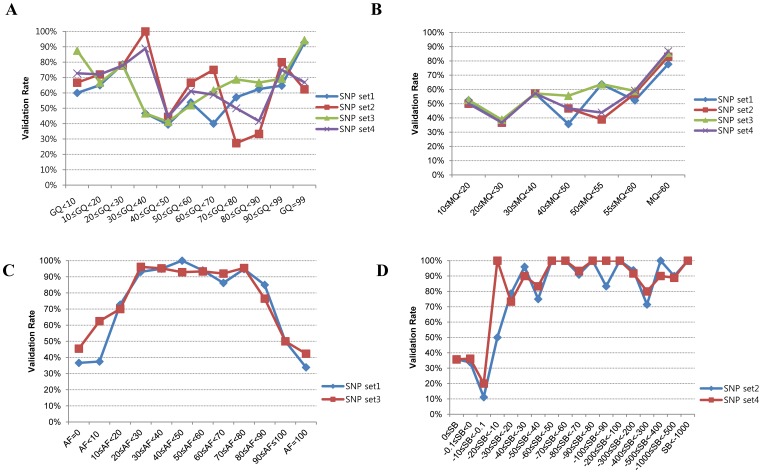
Evaluation of analysis parameters for improving validation rates. (A) Distribution of validation rates according to genotype quality (GQ) values. (B) Distribution of validation rates according to mapping quality (MQ) values. (C) Distribution of validation rates according to alternate allele forward (AF) percent for SAMtools (SNP set1 and SNP set3). (D) Distribution of validation rates according to strand bias (SB) values for GATK (SNP set2 and SNP set4).

Even if the validation rate of variants was improved by the selection of common variants, it still fell short of research expectations. We also checked other possible parameters to see whether they were good for selecting and validating SNPs. We did not find a correlation between GQ and validation rate (Figure 4A). In the case of MQ, the validation rate was greater than 80% when variants were filtered with values greater than 58 by SAMtools (SNP set1 and SNP set3) and GATK (SNP set2 and SNP set4) (Figure 4B). The SB of NGS data showed discriminating power between the variants that passed validation and those that failed validation. These results showed over a 92% validation rate when alternate allele forward [AF]≥20 and AF<80 in SAMtools (Figure 4C) and SB<–10 in GATK (Figure 4D).

**Figure 5:**
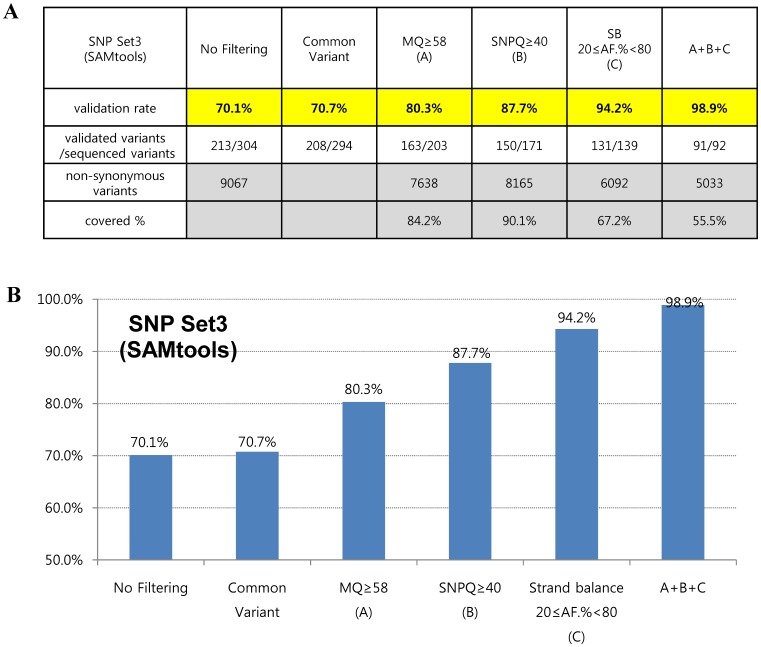
Validation rates according to suggested cutoff values of parameters are shown in table (A) and graph (B) form using the SAMtools algorithm after realignment and recalibration. A+B+C: filtered variants together with suggested values of SNPQ, GQ, and SB.

**Figure 6:**
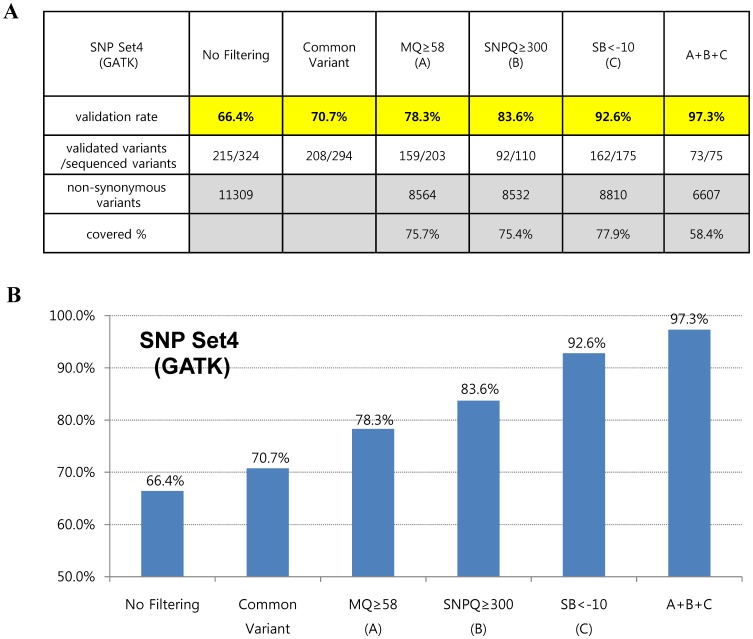
Validation rates according to suggested cutoff values of parameters are shown in table (A) and graph (B) form using the GATK algorithm after realignment and recalibration. A+B+C: filtered variants together with suggested values of SNPQ, GQ, and SB.

To further assess the accuracy of variant calling, we applied filtering operations together with suggested values of MQ, SNPQ and SB. Interestingly, the filtered SNVs were not false-positives for the most part, showing validation rates of 98.9% and 97.3% in SAMtools (SNP set3) and GATK (SNP set4), respectively (Figures 5 and 6). In addition, they covered more than half of all non-synonymous variants in SAMtools (5,033/9,067) and GATK (6,607/11,309), respectively (Figures 5 and 6).

## Discussion

We determined comprehensive cutoff values for calling SNVs found in NGS data. Our results improve the process of validation, using various SNP analysis sets and sub-categorized parameters. For clinical application, it is important to reduce the number of variants that require confirmation by Sanger sequencing (lower the false-positive rate) to maintain an acceptable cost-benefit ratio [16]. The comprehensive cutoff values provided herein enable one to discover somatic and germline mutations with low rates of false-positive variants, and then to implement clinical testing immediately.

Figures 5 and 6 show interesting findings according to suggested values of analysis parameters in our evaluation. We first found that common variants (variants that were filtered by both the SAMtools and GATK algorithms) led to a slight increase in the validation rate. When SNVs were filtered with an MQ above 58 in both SAMtools and GATK, the validation rates increased to 80.3% and 78.3%, respectively. In the case of SNPQ, the validation rate increased to 87.7% and 83.6% when SNVs were filtered with 40 in SAMtools and 300 in GATK. Interestingly, when SNVs were filtered with an SB of 20≤AF.%<80 in SAMtools and SB<–10 in GATK, the validation rate increased significantly, to 94.2% and 92.6%, respectively. Moreover, when we filtered with MQ, SNPQ, and SB at the same time with suggested values, the filtered SNVs were not false-positives for the most part, showing validation rates of 98.9% and 97.3% with SAMtools and GATK, respectively (Figures 5 and 6). We performed Sanger sequencing to validate two exome data sets produced with different capture kits and the NGS platform (Sureselect Library exome kit and Illumina HiSeq2000) and compared them to the results of this study. We sequenced 40 and 46 variants (from the two data sets, respectively) selected randomly among non-synonymous variants using our suggested MQ, SNPQ and SB values (Figures 5 and 6), and then examined the validation rate. The results revealed that 39 (of 40 variants) and 46 (of 46 variants) were validated for the GATK algorithm, and 38 (of 39 variants) and 41 (of 41 variants) were validated for the SAMtools algorithm. Therefore, we confirmed the high validation rate (with 99% accuracy) observed in other data sets.

We applied the most frequently used tools, the SAMtools and GATK programs, to identify variants. Each has different parameters and quality scores for variant detection. Using the SNP data of set3 and set4, there were 294 common variants and 208 variants was validated with a 70.7% validation rate (Figure 3). There were 10 specific variants in SNP set3 (SAMtools) and 30 in SNP set4 (GATK). When we examined caller-specific variants, the validation rate was very low, 30% (12/40). The choice of software tool clearly impacts the mutations identified, and the FDR of mutations found with the consensus of SAMtools, GATK, and Somatic SNiPer is very low, much lower than those found with specific callers [10]. We confirmed that common variants produced the highest enrichment of true-positive variants. Based on our analyses, the validation rate for SAMtools may be higher than that for GATK. However, this cannot be concluded because we used different suggested cutoff values and there are different covered regions between SAMtools and GATK. If multiple analyses are indispensable, then the caller-specific recommendations suggested in this study should be used to improve the validation rate.

Our results also suggest that SB is a key parameter that increases the validation rate. SB is observed either when the majority of sequence reads originate from only one DNA strand, or when variant bases occur preferentially on one strand compared to the other. Inaccurate base calls are more likely to cluster on one strand of DNA [17]. Thus, reads from both forward and reverse strands should be considered for making accurate variant calls and reducing errors. Appropriate filtering cutoff values should be developed to minimize errors due to SB. We suggest first stetting a confident cutoff value for the distribution of reads on the forward and reverse strands to improve the validation.

It is generally believed that SNVs with high read depth are more reliable than SNPs with low read depth [18]. However, we found that high-depth SNPs tended to be validated less than low-depth SNPs (Figure 2C). We categorized 45 combinations according to TD and SNPQ, and examined the validation rate. We found a positive correlation between TD and SNPQ values (Table 1). For example, only high TD variants with high SNPQ were validated. There may have been a close connection between TD and SNPQ when variants were called exactly as SNVs. These results suggest that it is difficult to select a variant with only a high TD value without a high SNPQ value.

Various quality parameters should be assessed and compared to those established during validation [19]. However, the cutoff values for each parameter have been unclear. In this study, we examined GQ and MQ as well as TD and SNPQ. For GQ, we identified no significant values for validation with either the SAMtools or GATK programs, but we did identify significant cutoff values for MQ. We also found that the SNVs that failed validation were not distributed in sequence repeat regions or copy number variation regions by searching the established Database of Genomic Variants (http://projects.tcag.ca/variation/). In addition, we examined the association between alternation of depth and validation rates. Whether the alternation was hetero (15–80%) or homo, we could not see a difference in the validation rate (data not shown).

In conclusion, we systematically examined important factors that could improve the validation rate in NGS data. These parameters were (1) SNPQ (more than 40 in SAMtools and more than 300 in GATK), (2) realignment and recalibration, (3) common variant analysis, (4) MQ greater than 58 and (5) SB (20≤AF.%<80 in SAMtools and SB<–10 in GATK). Our results have important implications for understanding the accuracy and completeness of variant calling in NGS data. This detailed and systematic study provides some guidelines for improving validation rates, saving time and lowering cost in NGS analysis. Furthermore, our method is applicable to diagnostic algorithms or therapeutic target selection because it can distinguish between true mutations and false-positives.

## Supporting Information

Figure S1 **Quality control for 30 exome-sequencing data sets.** Minimum (Min), first quartile (Q1), median, third quartile (Q3) and maximum (Max) values of SNP quality (SNPQ) were analyzed in 9 groups to evaluate the congruence of 30 exome data sets. There was no significant difference in SNPQ value pattern.
(TIF)

Figure S2 **Sanger sequencing traces for both a true-positive and a false-positive variant call.** (A) Example of a true-positive variant: chr1∶45797505 identified in next-generation sequencing (NGS) data (left) and confirmed by Sanger sequencing (right). (B) Example of a false-positive variant: chr1∶152280265 identified in NGS data (left) and confirmed by Sanger sequencing (right).
(TIF)

Dataset S1 **Data set for 348 variants generated from SNP set1 pipeline.**
(XLS)

Dataset S2 **Data set for 336 variants generated from SNP set2 pipeline.**
(XLS)

Dataset S3 **Data set for 304 variants generated from SNP set3 pipeline.**
(XLS)

Dataset S4 **Data set for 324 variants generated from SNP set4 pipeline.**
(XLS)
