# Supplementary figures and images for: Comprehensive Analysis to Improve the Validation Rate for Single Nucleotide Variants Detected by Next-Generation Sequencing
Source: PLoS One. 2014 Jan 29;9(1):e86664. doi: 10.1371/journal.pone.0086664 (PMC3906084; doi:10.1371/journal.pone.0086664)

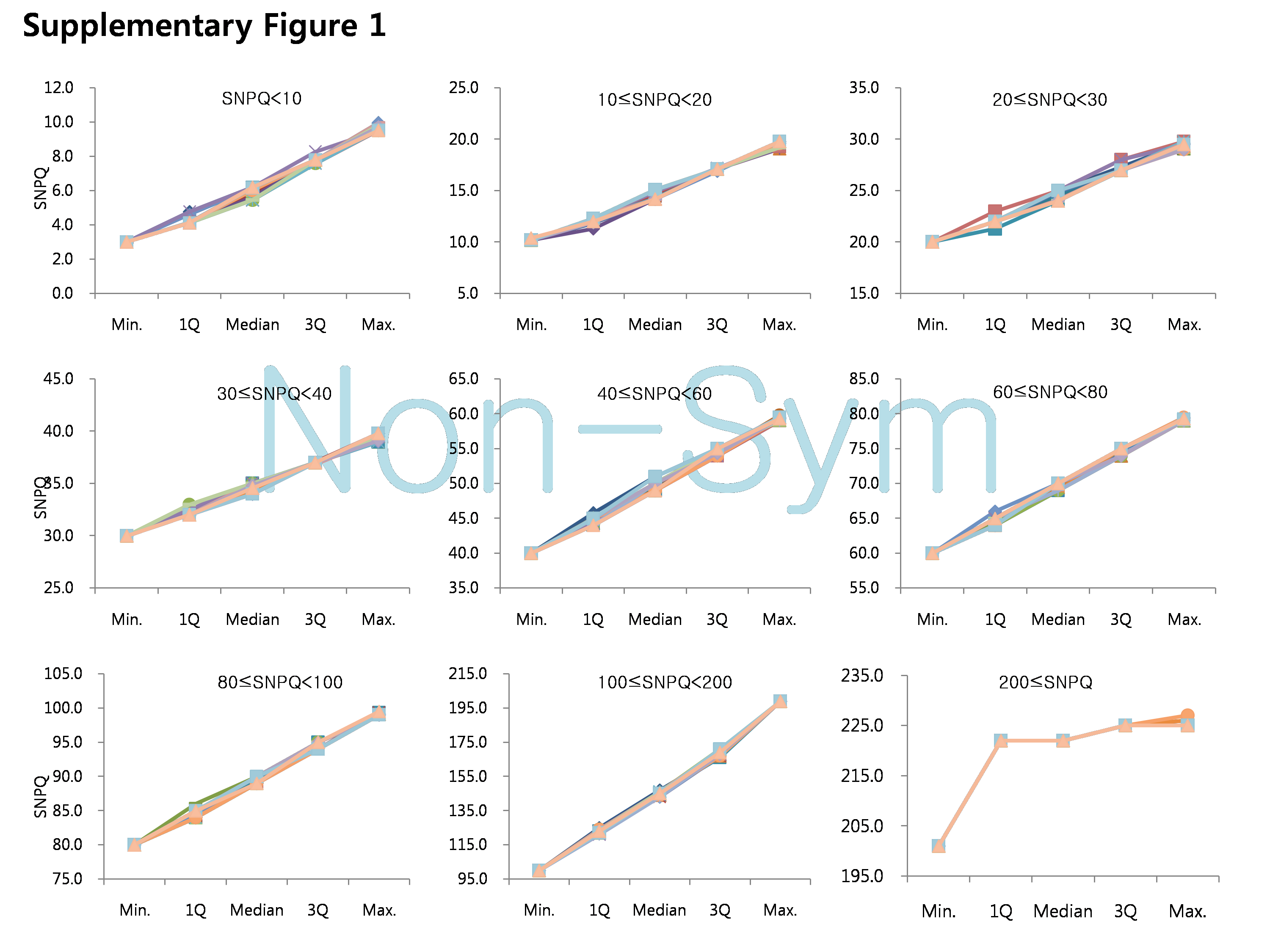

Supplement: Figure S1 — Quality control for 30 exome-sequencing data sets. Minimum (Min), first quartile (Q1), median, third quartile (Q3) and maximum (Max) values of SNP quality (SNPQ) were analyzed in 9 groups to evaluate the congruence of 30 exome data sets. There was no significant difference in SNPQ value pattern. (TIF) [file pone.0086664.s001.tif]

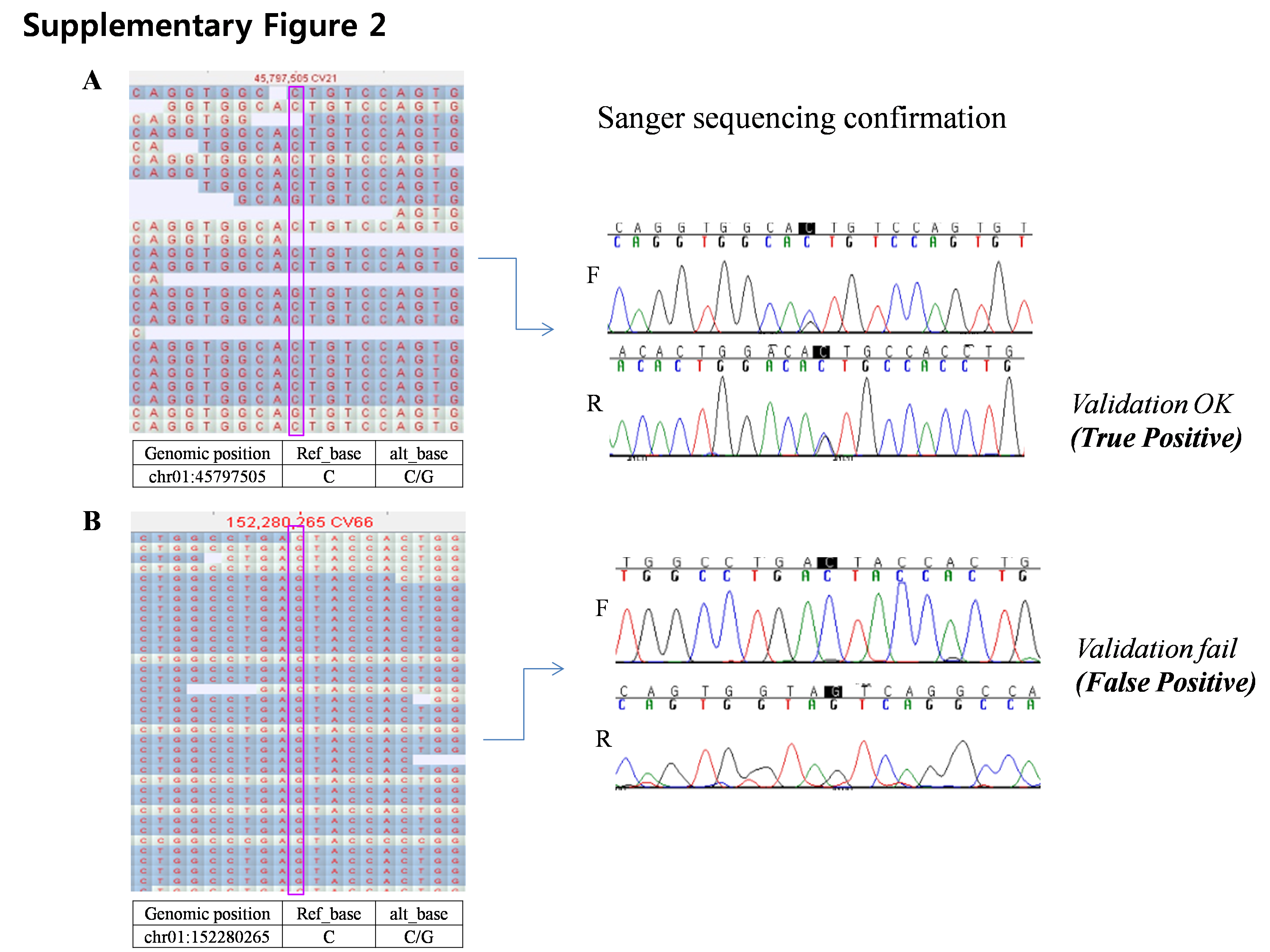

Supplement: Figure S2 — Sanger sequencing traces for both a true-positive and a false-positive variant call. (A) Example of a true-positive variant: chr1∶45797505 identified in next-generation sequencing (NGS) data (left) and confirmed by Sanger sequencing (right). (B) Example of a false-positive variant: chr1∶152280265 identified in NGS data (left) and confirmed by Sanger sequencing (right). (TIF) [file pone.0086664.s002.tif]
